# A Yellow Fever Scare in Texas

**Published:** 1899-02

**Authors:** 


### A Yellow Fever Scare in Texas.

FROM A REFUGEE'S DAIRY.

Saturday, October 9, 1897:

It was 9 o'clock, and through the house rang out the alarm of yellow fever! Guiteras, the *expert,* sent here by the Marine Hospital Service, had declared that yellow fever actually existed in Galveston.

Sunday, October 10, 1897:

I resolved to leave today, feeling no desire to be a culture tube for the *bacillus icteroideus.*

I got out on time at 7:15 a. m., on the Santa Fe train. Met several others with the same determined notion not to propagate germs in their carcasses. At every station we were met by officers telling us "move on." Every time our train stopped people, claiming to be afraid of the fever, would collect around the depot to get a glimpse of a yellow fever train. The human curiosity is a peculiar thing, treacherous, misleading—even malicious.

At Sealy it was great sport to watch negroes trying to get off. They had not been to Galveston at all, in fact had taken the down train that morning, and, after going one station, found they could not go farther, so they returned on the next train; but the scared, nonsensical citizens would not let any one get off; they had "quarantined against the world!" The darkies were not to be outdone; so, just as soon as the train got under good headway, one determined fellow jumped off and away sped the train. It was a beautiful, yet ludicrous, spectacle to see that darkey running through the grass and weeds with just *acres* of people after him. Men, old and young; boys of all kinds; coons, Mexicans, anything that could run and shout, took out after this unfortunate fellow. He was young, strong, swift, determined, but his pursuers were equally determined and were———. We were denied the *denouement*—the train sped out of sight to meet a few miles farther on another set of terrified, curiosity-excited citizens, who told by their looks, as well as by their guns and clubs, that they had no home for refugees.

Nothing of special importance occurred until we reached the suburbs of Brenham, a town that had suffered all the horrors of an epidemic years ago. The people had not forgotten the destruction of their little town, the demoralization of business, the newly-made graves, the separation of loved ones—yea, more, the anguish, the blighting destruction of an epidemic which almost decimated the town. Neither had they forgotten this, nor did they intend to have another if a rigid quarantine—more than rigidly enforced—could prevent it. Here, too, we were met by the town *en masse,* one-half of whom seemed to be guards, judging from guns, clubs, and "cussing," which characterized most of them. A white man jumped off, but a committee of one thousand, plus, of the most mottled populace that could be gotten together, including dogs, waited on him, though not daring to touch him, and literally forced him onto the train. They would have shot him with long range guns had he not gotten on. The invitation was too great—he got on! This all occurred before the train reached the depot. One very amusing thing was to see how authoritatively an officiously-inclined person can act when vested with only a semblance of power. One guard, standing in front of our coach, was gesticulating like an Indian and swearing like a trooper, even daring someone to get off; but still he grew more furious and picked up about one-fourth of a railroad tie and brandished it like a Hercules. Everybody laughed at him, and as the train passed some one of the boys pitched a handful of germs into his face and yelled, "Ta, ta, bud!"

The same rigid quarantine was met at every place, and there were boys on board who lived in South Texas but could not get off. They were carried free, of course.

"You know the rest."

—*H., in University Medical.*

[Do we need anything in the way of sanitary legislation? Oh, no,—not at all.—Ed.]

